# Supplementary material for: Modeling non-pharmaceutical interventions in the COVID-19 pandemic with survey-based simulations
Source: PLoS One. 2021 Oct 28;16(10):e0259108. doi: 10.1371/journal.pone.0259108 (PMC8553158; doi:10.1371/journal.pone.0259108)
Supplement: S1 Table — * At the time of conducting our simulation experiments, we used the most recent values listed in the latest version of Covasim and the associated article. Unfortunately, these values have changed (slightly) in the final release of Covasim. Nevertheless, we do not expect these changes to have a significant impact on our model results [34, 36, 47, 48]. (PDF) [file pone.0259108.s001.pdf]

S1 Table. Values and data sources of simulation parameters.

Parameter	Value	Source
Hours worked on weekdays	agent-specific	[34]
Age	agent-specific	[34]
Gender	agent-specific	[34]
Federal state	agent-specific	[34]
NACE-section	agent-specific	[34]
Household weight	household-specific	[34]
Enrollment at the university	agent-specific	[34]
Daily hours at stores	agent-specific	[34]
Daily hours at kindergarten	5	Assumption
Daily hours at school	5	Assumption
Daily hours at universities	4	Assumption
Kindergarten age	0 - 5	Assumption
School age	6 - 19	Assumption
Supermarkets per agents	1/1000	Assumption
Colleagues per work place	10	Assumption
Number of supermarkets an agent visits	2	Assumption
Number of Universities	1	Assumption
Days until an agent cures symptoms at home	1	Assumption
Duration of household quarantine in days	14	Assumption
Daytime	8 - 21	Assumption
Probability of developing symptoms at age 0-9	0.5	[36]
Probability of developing symptoms at age 10-19	0.55	[36]
Probability of developing symptoms at age 20-29	0.6	[36]
Probability of developing symptoms at age 30-39	0.65	[36]
Probability of developing symptoms at age 40-49	0.7	[36]
Probability of developing symptoms at age 50-59	0.75	[36]
Probability of developing symptoms at age 60-69	0.8	[36]
Probability of developing symptoms at age 70-79	0.85	[36]
Probability of developing symptoms at age > 80	0.9	[36]
Days from exposed to presymp. infectious	lognormal(4.6, 4.8)*	[36]
Days from presymp. infectious to (a)symp. infectious	lognormal(1, 1)*	[36]
Days from (a)symptomatic to recovered	lognormal(8, 2)	[36]
Average class size Baden-Wuerttemberg	22	[47]
Average class size Bavaria	23	[47]
Average class size Hamburg	25	[47]
Average class size Saarland	24	[47]
Average kindergarten group size in Baden-Wuerttemberg	15	[48]
Average kindergarten group size in Bavaria	18	[48]
Average kindergarten group size in Hamburg	18	[48]
Average kindergarten group size in Saarland	18	[48]

* At the time of conducting our simulation experiments, we used the most recent values listed in the latest version of Covasim and the associated article. Unfortunately, these values have changed (slightly) in the final release of Covasim. Nevertheless, we do not expect these changes to have a significant impact on our model results.
