# Supplementary material for: Modeling non-pharmaceutical interventions in the COVID-19 pandemic with survey-based simulations
Source: PLoS One. 2021 Oct 28;16(10):e0259108. doi: 10.1371/journal.pone.0259108 (PMC8553158; doi:10.1371/journal.pone.0259108)
Supplement: S2 Table — (PDF) [file pone.0259108.s002.pdf]

S2 Table. Descriptive statistics on federal states.

Variable	Baden-Wuertt.	Bavaria	Hamburg	Saarland	Source
GDP 2019 in billions €	524.33	632.90	123.27	36.25	[49]
Inhabitants per km^2 2018	310	185	2438	385	[50]
Average age in agent pop.	46.153958	45.791286	45.164004	44.727600	[34]
Average household size in agent pop.	2.791733	2.605203	2.735912	2.513871	[34]
Average work hours in agent pop.	3.091075	3.106836	2.696887	3.082421	[34]
Most frequent NACE-section in agent pop.	C (14.62%)	C (13.02%)	Q (7.94%)	Q (12.72%)	[34]
Men in agent pop.	48.33%	48.47%	44.86%	46.44%	[34]
