# Supplementary material for: Modeling non-pharmaceutical interventions in the COVID-19 pandemic with survey-based simulations
Source: PLoS One. 2021 Oct 28;16(10):e0259108. doi: 10.1371/journal.pone.0259108 (PMC8553158; doi:10.1371/journal.pone.0259108)
Supplement: S3 Table — gives the percentages to which the work hours were reduced in the second quarter (April, May, June) of 2020 as compared to 2019 per NACE-section. We assume that the differences between 2020 and 2019 are mainly caused by the COVID-19 pandemic. The values are taken from [51]. (PDF) [file pone.0259108.s003.pdf]

S3 Table. Reduction of work hours per NACE-section.

NACE-section	% Work hour reduction
A Agriculture, Forestry and Fishing	-4.7
B Mining and Quarrying	-2.8
C Manufacturing	-9.9
D Electricity, Gas, Steam and Air Conditioning ...	-0.6
E Water Supply; Sewerage, Waste Management and ...	-1.5
F Construction	-1.5
G Wholesale and Retail Trade; Repair of Motor V...	-7.9
H Transportation and Storage	-5.6
I Accommodation and Food Service Activities	-32.3
J Information and Communication	-4.0
K Financial and Insurance Activities	-0.8
L Real Estate Activities	-2.5
M Professional, Scientific and Technical Activi...	-4.4
N Administrative and Support Service Activities	-9.7
O Public Administration and Defence; Compulsory...	0.0
P Education	-1.5
Q Human Health and Social Work Activities	-2.0
R Arts, Entertainment and Recreation	-15.5
S Other Service Activities	-6.3
T Activities of Households as Employers; Undiff...	-4.7
U Activities of Extraterritorial Organisations ...	-4.7

S3 Table gives the percentages to which the work hours were reduced in the second quarter (April, May, June) of 2020 as compared to 2019 per NACE-section. We assume that the differences between 2020 and 2019 are mainly caused by the COVID-19 pandemic. The values are taken from [51].
