# Supplementary material for: Modeling non-pharmaceutical interventions in the COVID-19 pandemic with survey-based simulations
Source: PLoS One. 2021 Oct 28;16(10):e0259108. doi: 10.1371/journal.pone.0259108 (PMC8553158; doi:10.1371/journal.pone.0259108)
Supplement: S4 Table — The column “Normal” gives the share of people frequently working at home office per NACE-division. The column “Maximum” gives the share to which the work could be done in home office i.e. the maximum capacity of working from home per NACE-division. We interpret all values as share of work hours per NACE-division. We assume that in the total lock- and shutdown in the first wave of COVID-19 in Germany 2020 the full capacity of working from home was exploited in each NACE-division. All values are taken from [35]. (PDF) [file pone.0259108.s004.pdf]

## S4 Table. Amount of working from home per NACE-divisions.

| NACE-division | Normal | Maximum |
|---|---|---|
| 1 Crop and animal production, hunting and relat... | 0.070 | 0.372 |
| 2 Forestry and logging | 0.077 | 0.391 |
| 3 Fishing and aquaculture | 0.074 | 0.376 |
| 5 Mining of coal and lignite | 0.047 | 0.394 |
| 6 Extraction of crude petroleum and natural gas | 0.079 | 0.531 |
| 7 Mining of metal ores | 0.045 | 0.393 |
| 8 Other mining and quarrying | 0.047 | 0.390 |
| 9 Mining support service activities | 0.058 | 0.461 |
| 10 Manufacture of food products | 0.061 | 0.418 |
| 11 Manufacture of beverages | 0.086 | 0.494 |
| 12 Manufacture of tobacco products | 0.085 | 0.546 |
| 13 Manufacture of textiles | 0.070 | 0.579 |
| 14 Manufacture of wearing apparel | 0.099 | 0.654 |
| 15 Manufacture of leather and related products | 0.067 | 0.578 |
| 16 Manufacture of wood and of products of wood ... | 0.051 | 0.428 |
| 17 Manufacture of paper and paper products | 0.066 | 0.586 |
| 18 Printing and reproduction of recorded media | 0.074 | 0.634 |
| 19 Manufacture of coke and refined petroleum pr... | 0.085 | 0.612 |
| 20 Manufacture of chemicals and chemical products | 0.082 | 0.608 |
| 21 Manufacture of basic pharmaceutical products... | 0.086 | 0.626 |
| 22 Manufacture of rubber and plastic products | 0.059 | 0.483 |
| 23 Manufacture of other non-metallic mineral pr... | 0.060 | 0.436 |
| 24 Manufacture of basic metals | 0.051 | 0.434 |
| 25 Manufacture of fabricated metal products, ex... | 0.051 | 0.433 |
| 26 Manufacture of computer, electronic and opti... | 0.104 | 0.647 |
| 27 Manufacture of electrical equipment | 0.094 | 0.613 |
| 28 Manufacture of machinery and equipment n.e.c. | 0.078 | 0.554 |
| 29 Manufacture of motor vehicles, trailers and ... | 0.070 | 0.551 |
| 30 Manufacture of other transport equipment | 0.079 | 0.576 |
| 31 Manufacture of furniture | 0.057 | 0.456 |
| 32 Other manufacturing | 0.081 | 0.543 |
| 33 Repair and installation of machinery and equ... | 0.080 | 0.556 |
| 35 Electricity, gas, steam and air conditioning... | 0.111 | 0.684 |
| 36 Water collection, treatment and supply | 0.084 | 0.588 |
| 37 Sewerage | 0.066 | 0.502 |
| 38 Waste collection, treatment and disposal act... | 0.057 | 0.423 |
| 39 Remediation activities and other waste manag... | 0.070 | 0.481 |
| 41 Construction of buildings | 0.044 | 0.407 |
| 42 Civil engineering | 0.039 | 0.382 |
| 43 Specialised construction activities | 0.051 | 0.424 |
| 45 Wholesale and retail trade and repair of mot... | 0.070 | 0.521 |
| 46 Wholesale trade, except of motor vehicles an... | 0.126 | 0.641 |
| 47 Retail trade, except of motor vehicles and m... | 0.058 | 0.468 |
| 49 Land transport and transport via pipelines | 0.039 | 0.299 |
| 50 Water transport | 0.081 | 0.495 |
| 51 Air transport | 0.067 | 0.430 |
| 52 Warehousing and support activities for trans... | 0.062 | 0.427 |
| 53 Postal and courier activities | 0.053 | 0.385 |
| 55 Accommodation | 0.099 | 0.428 |
| 56 Food and beverage service activities | 0.083 | 0.390 |
| 58 Publishing activities | 0.167 | 0.836 |
| 59 Motion picture, video and television program... | 0.160 | 0.698 |
| 60 Programming and broadcasting activities | 0.177 | 0.798 |
| 61 Telecommunications | 0.133 | 0.735 |
| 62 Computer programming, consultancy and relate... | 0.187 | 0.874 |
| 63 Information service activities | 0.166 | 0.822 |
| 64 Financial service activities, except insuran... | 0.112 | 0.894 |
| 65 Insurance, reinsurance and pension funding, ... | 0.124 | 0.895 |
| 66 Activities auxiliary to financial services a... | 0.135 | 0.882 |
| 68 Real estate activities | 0.137 | 0.709 |
| 69 Legal and accounting activities | 0.109 | 0.876 |
| 70 Activities of head offices; management consu... | 0.139 | 0.765 |
| 71 Architectural and engineering activities; te... | 0.102 | 0.707 |
| 72 Scientific research and development | 0.175 | 0.706 |
| 73 Advertising and market research | 0.146 | 0.781 |
| 74 Other professional, scientific and technical... | 0.122 | 0.708 |
| 75 Veterinary activities | 0.039 | 0.436 |
| 77 Rental and leasing activities | 0.112 | 0.600 |
| 78 Employment activities | 0.060 | 0.444 |
| 79 Travel agency, tour operator and other reser... | 0.122 | 0.556 |
| 80 Security and investigation activities | 0.058 | 0.426 |
| 81 Services to buildings and landscape activities | 0.062 | 0.381 |
| 82 Office administrative, office support and ot... | 0.136 | 0.719 |
| 84 Public administration and defence; compulsor... | 0.108 | 0.693 |
| 85 Education | 0.302 | 0.725 |
| 86 Human health activities | 0.052 | 0.458 |
| 87 Residential care activities | 0.079 | 0.476 |
| 88 Social work activities without accommodation | 0.092 | 0.525 |
| 90 Creative, arts and entertainment activities | 0.168 | 0.635 |
| 91 Libraries, archives, museums and other cultu... | 0.134 | 0.659 |
| 92 Gambling and betting activities | 0.082 | 0.477 |
| 93 Sports activities and amusement and recreati... | 0.212 | 0.616 |
| 94 Activities of membership organisations | 0.154 | 0.700 |
| 95 Repair of computers and personal and househo... | 0.092 | 0.596 |
| 96 Other personal service activities | 0.057 | 0.418 |
| 97 Activities of households as employers of dom... | 0.108 | 0.547 |
| 98 Undifferentiated goods- and services-produci... | 0.055 | 0.406 |
| 99 Activities of extraterritorial organisations... | 0.098 | 0.631 |

The column ”Normal” gives the share of people frequently working at home office per NACE-division. The column ”Maximum” gives the share to which the work could be done in home office i.e. the maximum capacity of working from home per NACE-division. We interpret all values as share of work hours per NACE-division. We assume that in the total lock- and shutdown in the first wave of COVID-19 in Germany 2020 the full capacity of working from home was exploited in each NACE-division. All values are taken from [35].
