# Supplementary material for: Modeling non-pharmaceutical interventions in the COVID-19 pandemic with survey-based simulations
Source: PLoS One. 2021 Oct 28;16(10):e0259108. doi: 10.1371/journal.pone.0259108 (PMC8553158; doi:10.1371/journal.pone.0259108)
Supplement: S5 Table — shows the cumulative number of infected agents per 100,000 agents after 100 simulated days, averaged over 60 replications per state and scenario, along with the standard deviation and 95% confidence interval. (PDF) [file pone.0259108.s005.pdf]

S5 Table. Case numbers on day 100.

State	Scenario	Mean	Std	95%-CI lower	95%-CI upper
Baden-Wuerttemberg	Baseline	321.35	80.47	300.99	341.71
	No.Quarantine	1669.97	602.50	1517.52	1822.42
	Normal.HomeOffice	1004.59	418.80	898.62	1110.56
	Normal.Work.Hours	353.45	98.83	328.44	378.45
	Open.AllEduc	949.86	379.28	853.89	1045.83
	Open.Kinder	437.26	155.56	397.90	476.62
	Open.Schools	496.33	157.12	456.57	536.09
	Open.Schools_Kinder	848.91	324.64	766.76	931.05
	Open.Uni	368.50	134.77	334.40	402.60
Bavaria	Baseline	368.38	85.26	346.81	389.95
	No.Quarantine	1912.48	661.82	1745.02	2079.95
	Normal.HomeOffice	1396.66	599.24	1245.03	1548.29
	Normal.Work.Hours	462.00	106.76	434.98	489.01
	Open.AllEduc	1131.27	425.55	1023.60	1238.95
	Open.Kinder	654.61	218.29	599.38	709.85
	Open.Schools	692.21	225.66	635.11	749.31
	Open.Schools_Kinder	1081.66	373.56	987.14	1176.18
	Open.Uni	477.83	161.28	437.02	518.64
Hamburg	Baseline	281.30	42.65	270.51	292.09
	No.Quarantine	578.62	178.86	533.36	623.87
	Normal.HomeOffice	648.08	230.86	589.67	706.50
	Normal.Work.Hours	309.45	56.54	295.15	323.76
	Open.AllEduc	601.65	201.17	550.75	652.55
	Open.Kinder	364.58	115.12	335.46	393.71
	Open.Schools	438.25	129.48	405.49	471.02
	Open.Schools_Kinder	632.05	178.16	586.97	677.13
	Open.Uni	336.63	107.70	309.38	363.89
Saarland	Baseline	268.58	37.04	259.21	277.96
	No.Quarantine	564.00	158.31	523.94	604.06
	Normal.HomeOffice	524.60	155.91	485.15	564.05
	Normal.Work.Hours	310.35	56.93	295.95	324.76
	Open.AllEduc	511.67	178.66	466.46	556.87
	Open.Kinder	331.88	71.55	313.78	349.99
	Open.Schools	381.98	93.39	358.35	405.62
	Open.Schools_Kinder	480.97	165.64	439.06	522.88
	Open.Uni	274.55	49.39	262.05	287.05

S5 Table shows the cumulative number of infected agents per 100,000 agents after 100 simulated days, averaged over 60 replications per state and scenario, along with the standard deviation and 95% confidence interval.
