# Supplementary material for: Modeling non-pharmaceutical interventions in the COVID-19 pandemic with survey-based simulations
Source: PLoS One. 2021 Oct 28;16(10):e0259108. doi: 10.1371/journal.pone.0259108 (PMC8553158; doi:10.1371/journal.pone.0259108)
Supplement: S6 Table — shows the cumulative number of infected agents per 100,000 agents after 100 simulated days, averaged over 60 replications per state and scenario, as the difference from the corresponding baseline scenario, along with the standard deviation and 95 percent confidence interval. (PDF) [file pone.0259108.s006.pdf]

S6 Table. Case numbers on day 100 as the difference to baseline scenario.

State	Scenario	Mean	Std	95%-CI lower	95%-CI upper
Baden-Wuerttemberg	Baseline	0.00	80.47	-20.36	20.36
	No.Quarantine	1348.62	602.50	1196.17	1501.08
	Normal.HomeOffice	683.25	418.80	577.27	789.22
	Normal.Work.Hours	32.10	98.83	7.09	57.11
	Open.AllEduc	628.51	379.28	532.54	724.48
	Open.Kinder	115.92	155.56	76.55	155.28
	Open.Schools	174.98	157.12	135.23	214.74
	Open.Schools_Kinder	527.56	324.64	445.42	609.71
	Open.Uni	47.15	134.77	13.05	81.25
Bavaria	Baseline	0.00	85.26	-21.57	21.57
	No.Quarantine	1544.10	661.82	1376.64	1711.57
	Normal.HomeOffice	1028.28	599.24	876.65	1179.91
	Normal.Work.Hours	93.62	106.76	66.60	120.63
	Open.AllEduc	762.89	425.55	655.22	870.57
	Open.Kinder	286.23	218.29	231.00	341.47
	Open.Schools	323.83	225.66	266.73	380.93
	Open.Schools_Kinder	713.28	373.56	618.76	807.80
	Open.Uni	109.45	161.28	68.64	150.26
Hamburg	Baseline	0.00	42.65	-10.79	10.79
	No.Quarantine	297.32	178.86	252.06	342.57
	Normal.HomeOffice	366.78	230.86	308.36	425.20
	Normal.Work.Hours	28.15	56.54	13.84	42.46
	Open.AllEduc	320.35	201.17	269.45	371.25
	Open.Kinder	83.28	115.12	54.15	112.41
	Open.Schools	156.95	129.48	124.19	189.71
	Open.Schools_Kinder	350.75	178.16	305.67	395.83
	Open.Uni	55.33	107.70	28.08	82.58
Saarland	Baseline	0.00	37.04	-9.37	9.37
	No.Quarantine	295.41	158.31	255.36	335.47
	Normal.HomeOffice	256.02	155.91	216.56	295.47
	Normal.Work.Hours	41.77	56.93	27.36	56.17
	Open.AllEduc	243.08	178.66	197.87	288.29
	Open.Kinder	63.30	71.55	45.19	81.40
	Open.Schools	113.40	93.39	89.77	137.03
	Open.Schools_Kinder	212.38	165.64	170.47	254.29
	Open.Uni	5.97	49.39	-6.53	18.47

S6 Table shows the cumulative number of infected agents per 100,000 agents after 100 simulated days, averaged over 60 replications per state and scenario, as the difference from the corresponding baseline scenario, along with the standard deviation and 95 percent confidence interval.
