# Supplementary material for: Modeling non-pharmaceutical interventions in the COVID-19 pandemic with survey-based simulations
Source: PLoS One. 2021 Oct 28;16(10):e0259108. doi: 10.1371/journal.pone.0259108 (PMC8553158; doi:10.1371/journal.pone.0259108)
Supplement: S7 Table — shows the proportion of each age group in the cumulative number of infected agents on simulation day 100. (PDF) [file pone.0259108.s007.pdf]

S7 Table. Infected agents by age-group.

State	Scenario	0-29 Mean	0-29 Std	30-59 Mean	30-59 Std	60+ Mean	60+ Std
Baden-Wuerttemberg	Baseline	0.367	0.038	0.437	0.029	0.195	0.040
	No.Quarantine	0.388	0.016	0.462	0.015	0.150	0.019
	Normal.HomeOffice	0.364	0.015	0.485	0.020	0.150	0.025
	Normal.Work.Hours	0.360	0.031	0.438	0.035	0.203	0.042
	Open.AllEduc	0.456	0.035	0.434	0.016	0.110	0.038
	Open.Kinder	0.404	0.042	0.429	0.029	0.166	0.045
	Open.Schools	0.411	0.034	0.433	0.024	0.157	0.039
	Open.Schools_Kinder	0.454	0.033	0.433	0.018	0.112	0.041
	Open.Uni	0.368	0.034	0.440	0.026	0.192	0.043
Bayern	Baseline	0.329	0.026	0.466	0.031	0.205	0.043
	No.Quarantine	0.361	0.014	0.492	0.011	0.146	0.013
	Normal.HomeOffice	0.342	0.014	0.521	0.014	0.137	0.018
	Normal.Work.Hours	0.340	0.024	0.479	0.030	0.181	0.031
	Open.AllEduc	0.426	0.025	0.470	0.018	0.105	0.026
	Open.Kinder	0.389	0.032	0.475	0.020	0.136	0.032
	Open.Schools	0.389	0.030	0.476	0.022	0.135	0.033
	Open.Schools_Kinder	0.421	0.025	0.472	0.015	0.107	0.023
	Open.Uni	0.355	0.028	0.472	0.026	0.173	0.039
Hamburg	Baseline	0.373	0.038	0.425	0.050	0.202	0.048
	No.Quarantine	0.375	0.023	0.460	0.027	0.165	0.030
	Normal.HomeOffice	0.348	0.029	0.517	0.039	0.135	0.031
	Normal.Work.Hours	0.362	0.040	0.435	0.049	0.204	0.051
	Open.AllEduc	0.442	0.030	0.440	0.030	0.118	0.039
	Open.Kinder	0.390	0.040	0.435	0.041	0.175	0.056
	Open.Schools	0.425	0.041	0.426	0.029	0.149	0.048
	Open.Schools_Kinder	0.454	0.038	0.438	0.027	0.108	0.032
	Open.Uni	0.379	0.040	0.440	0.048	0.181	0.050
Saarland	Baseline	0.391	0.032	0.404	0.039	0.205	0.039
	No.Quarantine	0.411	0.031	0.422	0.028	0.167	0.026
	Normal.HomeOffice	0.385	0.027	0.454	0.034	0.160	0.025
	Normal.Work.Hours	0.397	0.038	0.415	0.039	0.187	0.046
	Open.AllEduc	0.470	0.043	0.392	0.024	0.139	0.036
	Open.Kinder	0.420	0.051	0.398	0.037	0.182	0.047
	Open.Schools	0.432	0.044	0.407	0.025	0.161	0.044
	Open.Schools_Kinder	0.468	0.042	0.398	0.025	0.135	0.035
	Open.Uni	0.392	0.036	0.408	0.038	0.200	0.045

S7 Table shows the proportion of each age group in the cumulative number of infected agents on simulation day 100.
