# Supplementary material for: Modeling non-pharmaceutical interventions in the COVID-19 pandemic with survey-based simulations
Source: PLoS One. 2021 Oct 28;16(10):e0259108. doi: 10.1371/journal.pone.0259108 (PMC8553158; doi:10.1371/journal.pone.0259108)
Supplement: S8 Table — shows the proportion of different vocational statuses per federal state in the population of agents. “None/other” are those agents who are neither working, studying, or of kindergarten or school age. “Working” means any agent who works more than zero hours per day and is not enrolled at a university. (PDF) [file pone.0259108.s008.pdf]

S8 Table. Share of vocational statuses per federal state.

state	Voc. status	Share of agents
Baden-Wuerttemberg	Working	0.435
	None/other	0.361
	Pupil	0.131
	Kindergarten kid	0.050
	Student	0.023
Bavaria	Working	0.446
	None/other	0.360
	Pupil	0.125
	Kindergarten kid	0.048
	Student	0.021
Saarland	Working	0.437
	None/other	0.377
	Pupil	0.126
	Kindergarten kid	0.045
	Student	0.014
Hamburg	Working	0.340
	None/other	0.432
	Pupil	0.143
	Kindergarten kid	0.051
	Student	0.035

S8 Table shows the proportion of different vocational statuses per federal state in the population of agents. "None/other" are those agents who are neither working, studying, or of kindergarten or school age. "Working" means any agent who works more than zero hours per day and is not enrolled at a university.
