# Supplementary material for: Modeling non-pharmaceutical interventions in the COVID-19 pandemic with survey-based simulations
Source: PLoS One. 2021 Oct 28;16(10):e0259108. doi: 10.1371/journal.pone.0259108 (PMC8553158; doi:10.1371/journal.pone.0259108)
Supplement: S9 Table — shows for each vocational status the average number of agents living in the same household by their vocational status. For instance, on average, an agent classified as a kindergarten child in Baden-Wuerttemberg lives in a household that contains 1.53 kindergarten children, 0.63 schoolchildren, 0.03 students, 1.4 employed persons, and 0.58 other persons. “None/other” are those agents who are neither working, studying, or of kindergarten or school age. “Working” means any agent who works more than zero hours per day and is not enrolled at an university. (PDF) [file pone.0259108.s009.pdf]

S9 Table. Household composition by vocational status and state.

state	Voc. status	Kinderg. kid	Pupil	Student	Working	None/other	Total
Baden-Wuerttemberg	None/other	0.08	0.20	0.02	0.35	1.56	2.21
	Kinderg. kid	1.53	0.63	0.03	1.40	0.58	4.17
	Pupil	0.24	2.01	0.03	1.47	0.53	4.29
	Student	0.06	0.29	1.19	1.07	0.30	2.90
	Working	0.16	0.45	0.06	1.71	0.29	2.68
Bavaria	None/other	0.08	0.17	0.02	0.38	1.49	2.14
	Kinderg. kid	1.40	0.36	0.02	1.28	0.61	3.67
	Pupil	0.14	1.84	0.03	1.48	0.48	3.98
	Student	0.05	0.26	1.12	0.77	0.41	2.60
	Working	0.14	0.42	0.04	1.59	0.31	2.49
Saarland	None/other	0.08	0.23	0.01	0.41	1.56	2.29
	Kinderg. kid	1.07	0.81	0.00	0.95	0.67	3.51
	Pupil	0.29	1.72	0.03	1.19	0.68	3.91
	Student	0.01	0.30	1.17	0.48	0.21	2.16
	Working	0.10	0.34	0.02	1.41	0.36	2.22
Hamburg	None/other	0.08	0.35	0.02	0.21	1.74	2.40
	Kinderg. kid	1.35	0.59	0.08	1.30	0.68	4.00
	Pupil	0.21	1.94	0.03	0.97	1.03	4.18
	Student	0.12	0.16	1.00	0.67	0.26	2.22
	Working	0.19	0.41	0.07	1.49	0.27	2.43

S9 Table shows for each vocational status the average number of agents living in the same household by their vocational status. For instance, on average, an agent classified as a kindergarten child in Baden-Wuerttemberg lives in a household that contains 1.53 kindergarten children, 0.63 schoolchildren, 0.03 students, 1.4 employed persons, and 0.58 other persons. "None/other" are those agents who are neither working, studying, or of kindergarten or school age. "Working" means any agent who works more than zero hours per day and is not enrolled at an university.
