# Supplementary figures and images for: Modeling non-pharmaceutical interventions in the COVID-19 pandemic with survey-based simulations
Source: PLoS One. 2021 Oct 28;16(10):e0259108. doi: 10.1371/journal.pone.0259108 (PMC8553158; doi:10.1371/journal.pone.0259108)

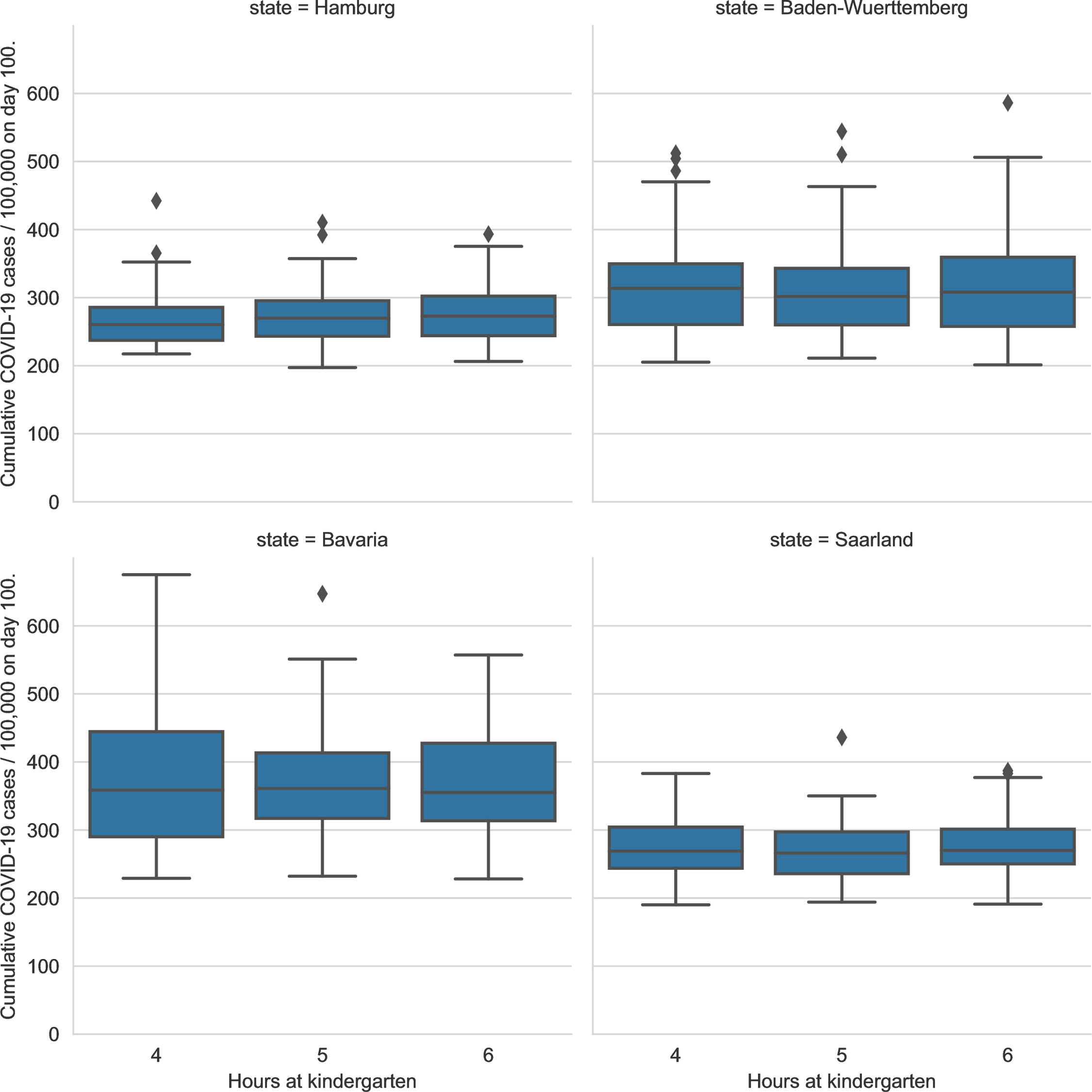

Supplement: S1 Fig — Compares the number of COVID-19 infections in the baseline scenario across different values for the daily hours at kindergarten. The value in the center is the one used in the main analysis. (TIF) [file pone.0259108.s010.tif]

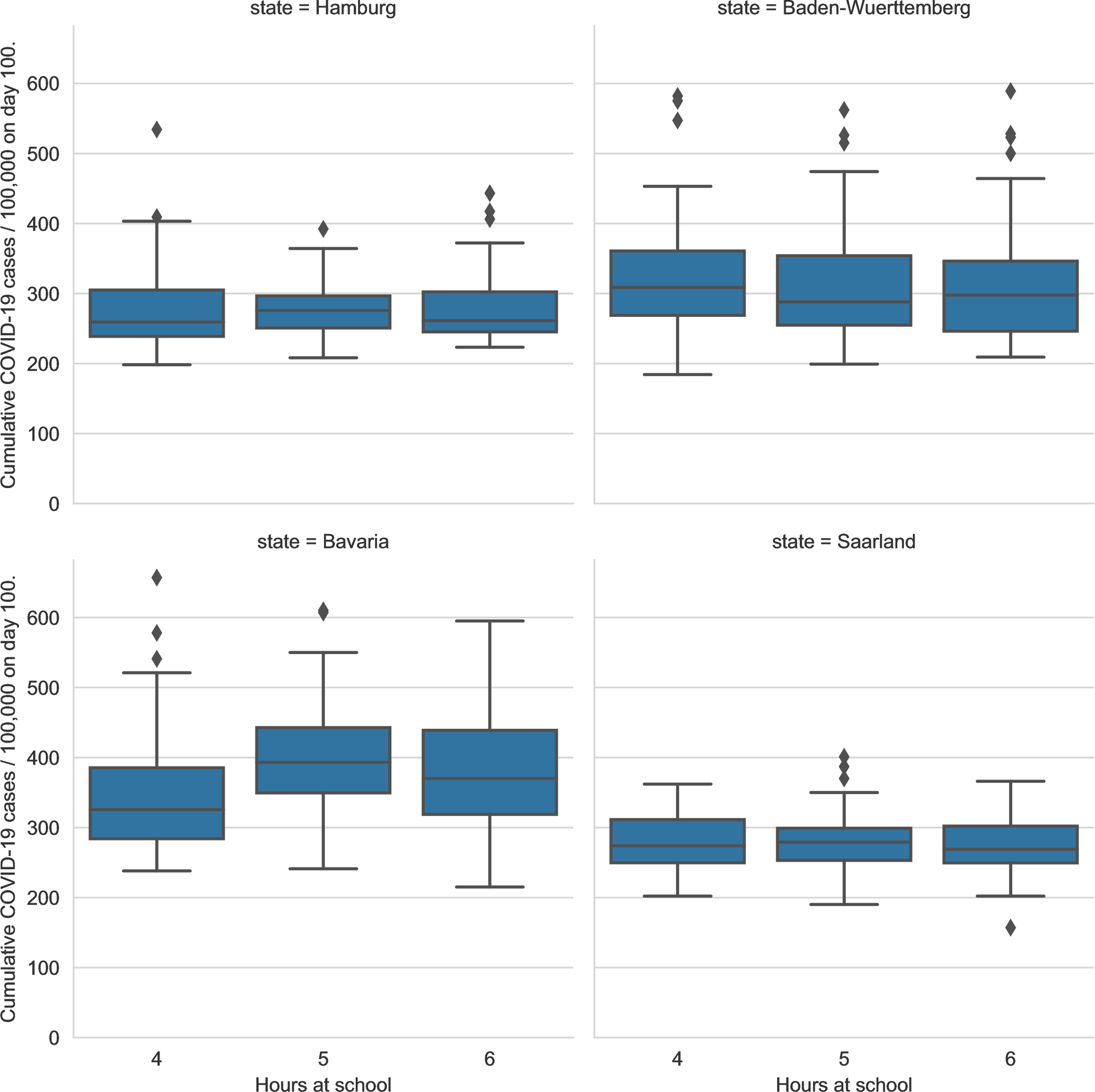

Supplement: S2 Fig — Compares the number of COVID-19 infections in the baseline scenario across different values for hours at school. The value in the center is the one used in the main analysis. (TIF) [file pone.0259108.s011.tif]

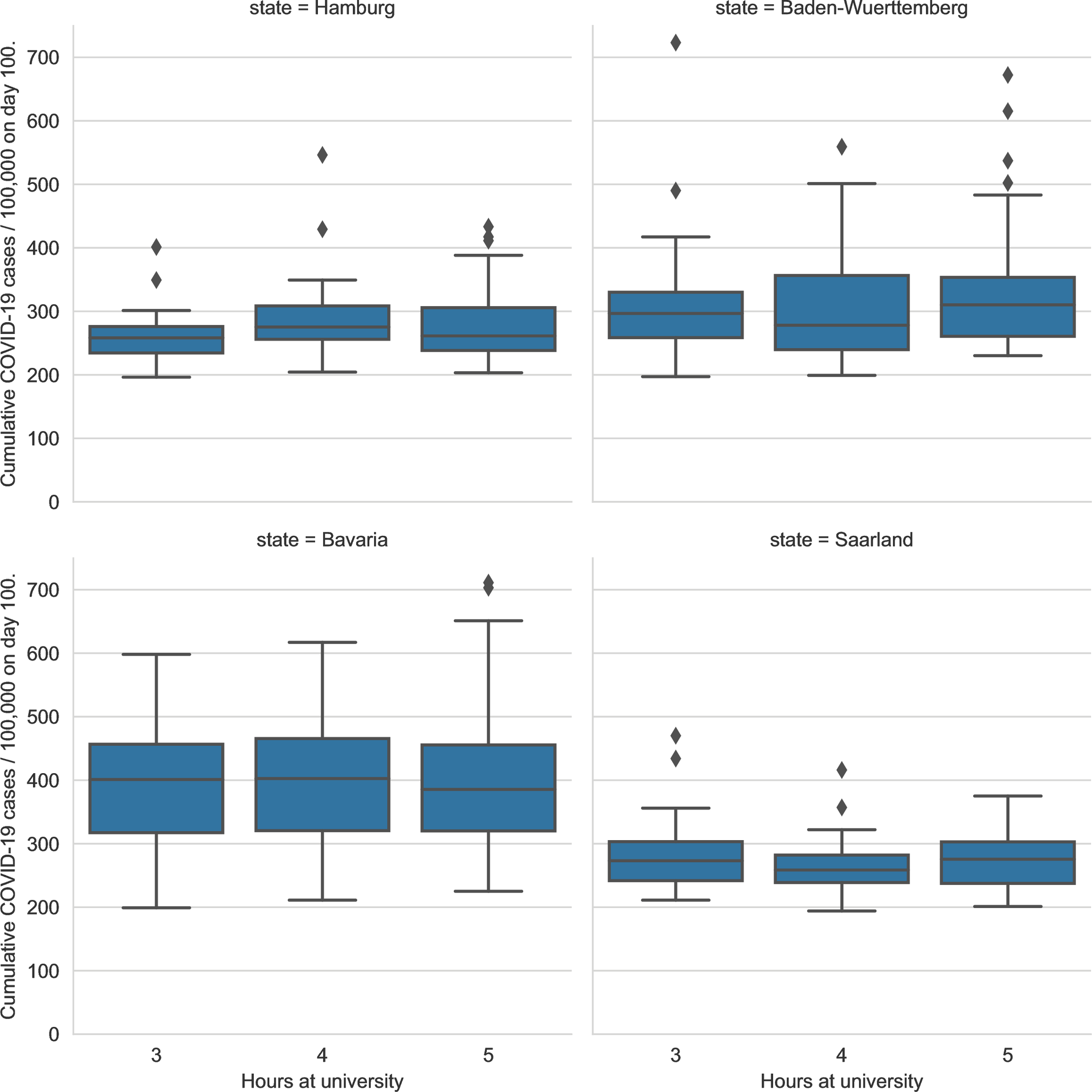

Supplement: S3 Fig — Compares the number of COVID-19 infections in the baseline scenario across different values for hours at university. The value in the center is the one used in the main analysis. (TIF) [file pone.0259108.s012.tif]

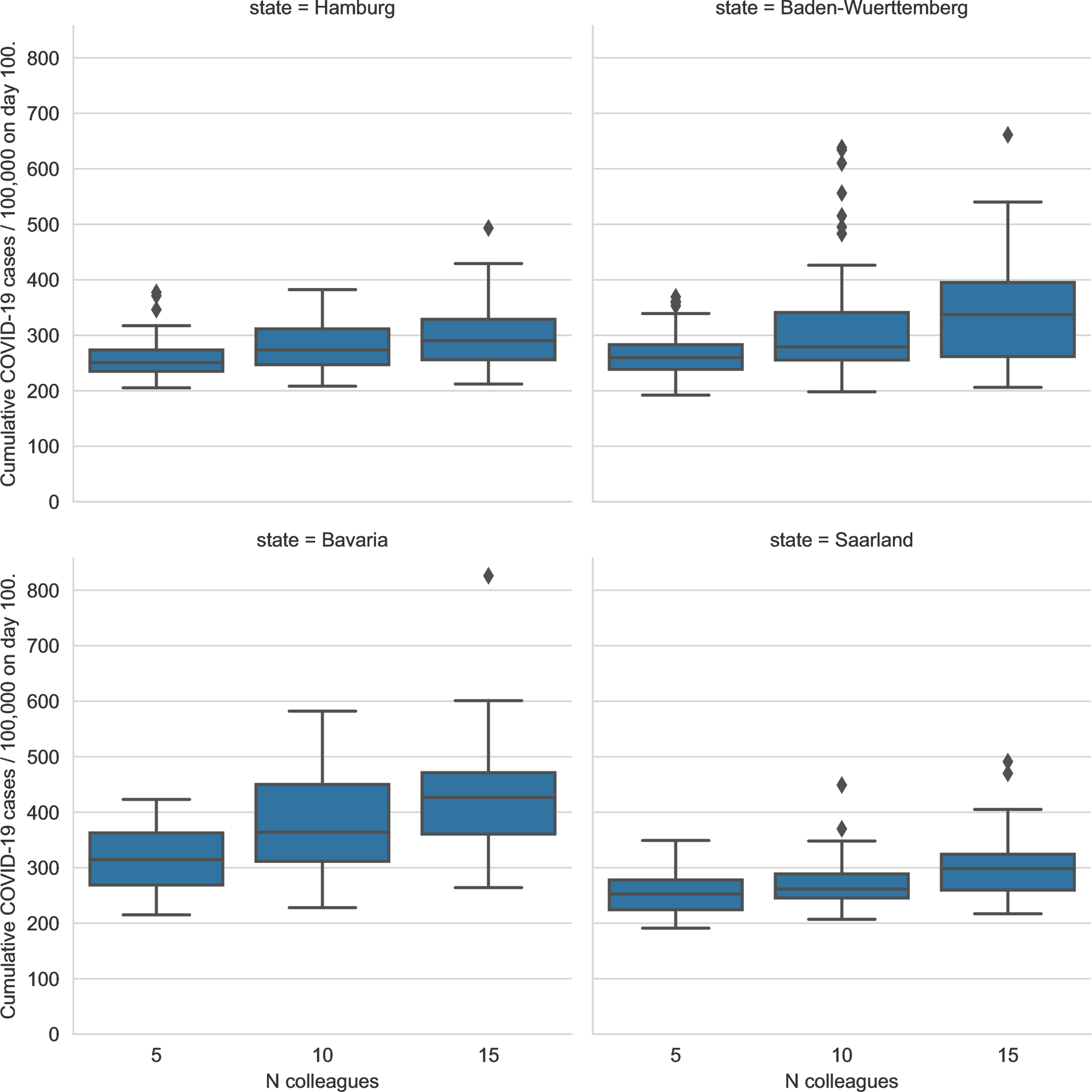

Supplement: S4 Fig — Compares the number of COVID-19 infections in the baseline scenario across different values for number of colleagues. The value in the center is the one used in the main analysis. (TIF) [file pone.0259108.s013.tif]

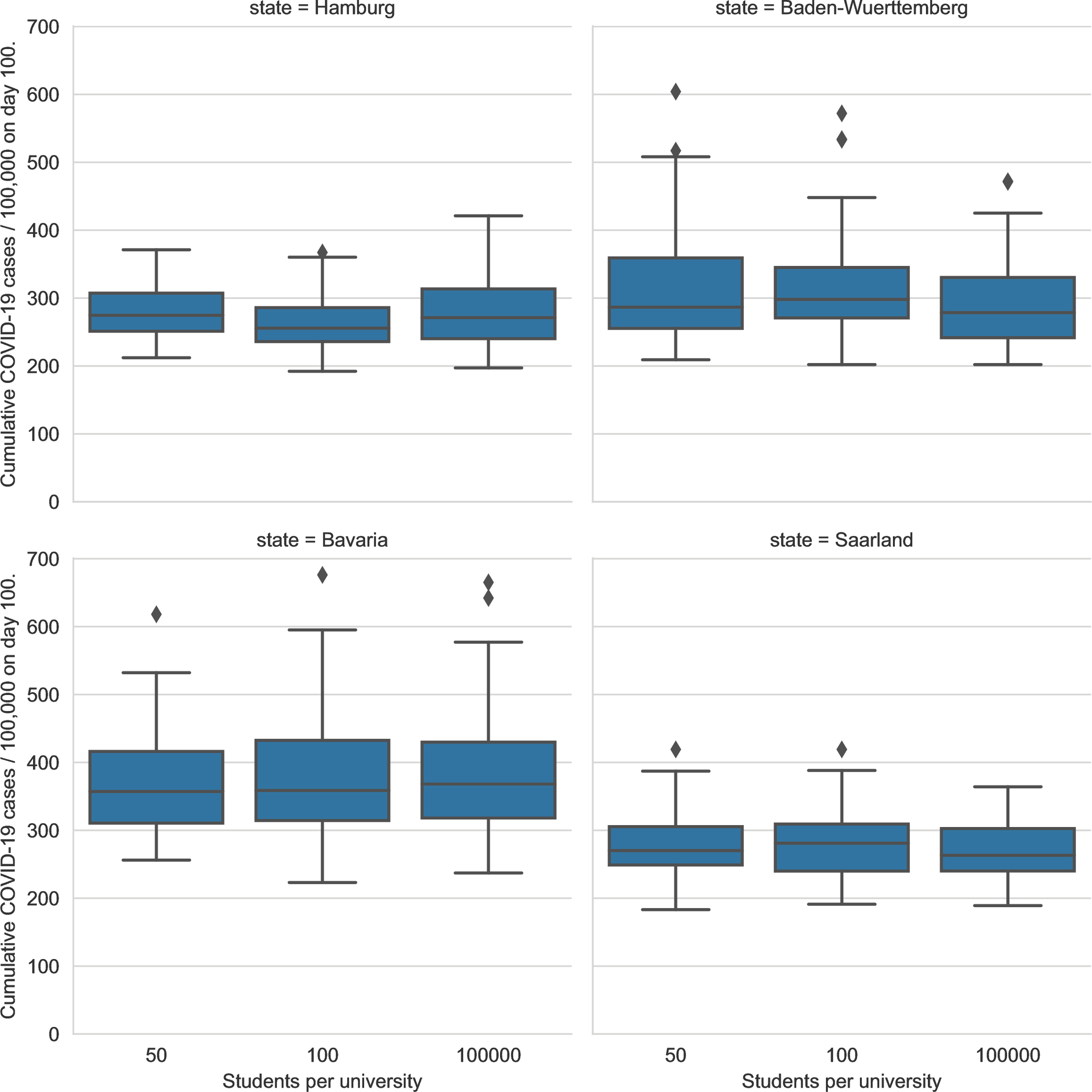

Supplement: S5 Fig — Compares the number of COVID-19 infections in the baseline scenario across different values for the maximum number of students per university. We used 100,000 in the main analysis, which means that there is only one university per simulated world. (TIF) [file pone.0259108.s014.tif]

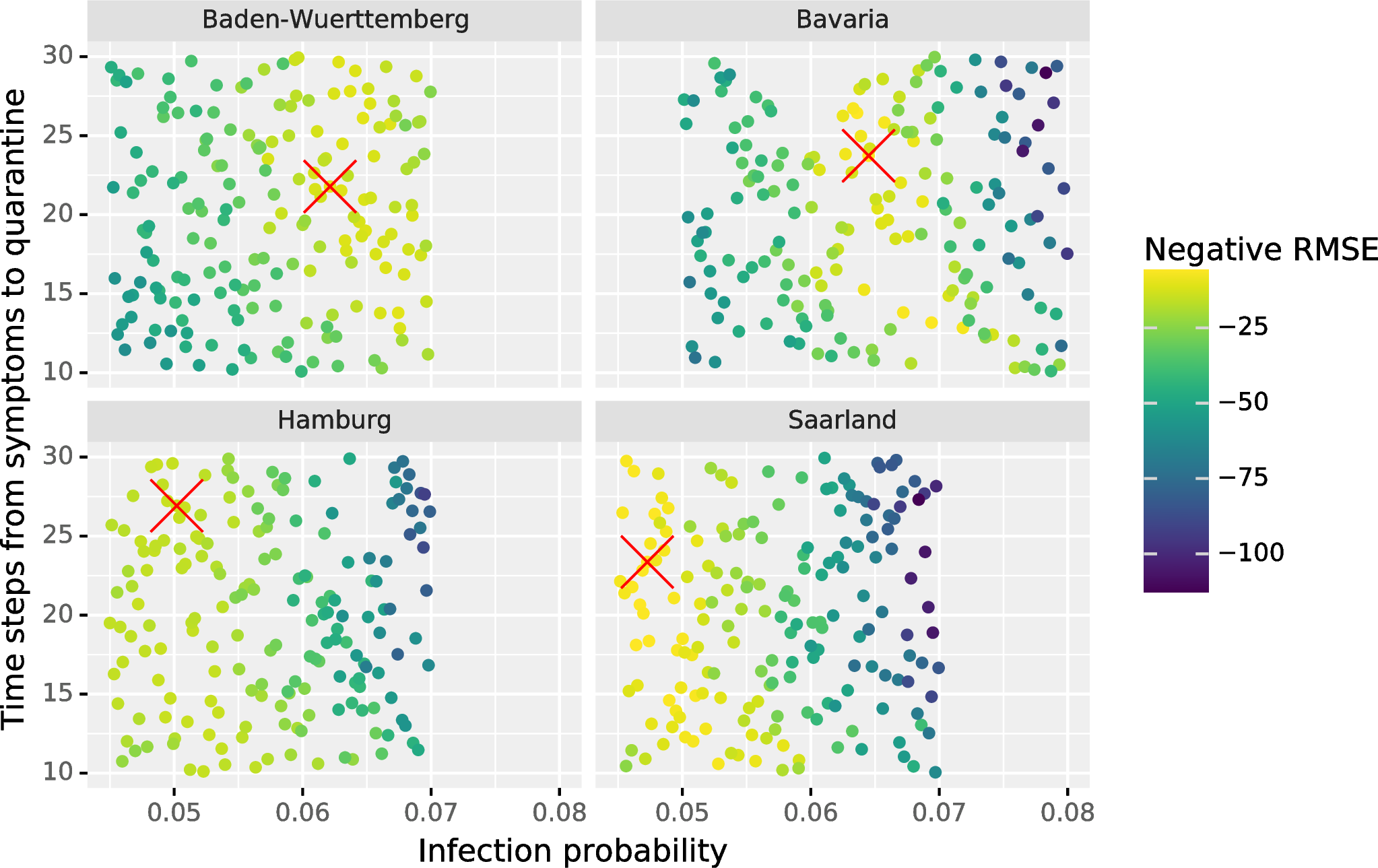

Supplement: S6 Fig — shows the tested combinations of the two calibrated parameters and the respective model fit when using the combination in the baseline model. The parameter combination that gives the best model fit is indicated by the cross. (TIF) [file pone.0259108.s015.tif]
